# XPC Promotes Pluripotency of Human Dental Pulp Cells through Regulation of Oct-4/Sox2/c-Myc

**DOI:** 10.1155/2016/3454876

**Published:** 2016-04-04

**Authors:** Lu Liu, Zhengjun Peng, Zhezhen Xu, Xi Wei

**Affiliations:** Operative Dentistry and Endodontics, Guanghua School of Stomatology, Affiliated Stomatological Hospital, Guangdong Province Key Laboratory of Stomatology, Sun Yat-sen University, Guangzhou, Guangdong 510055, China

## Abstract

*Introduction*. Xeroderma pigmentosum group C (XPC), essential component of multisubunit stem cell coactivator complex (SCC), functions as the critical factor modulating pluripotency and genome integrity through interaction with Oct-4/Sox2. However, its specific role in regulating pluripotency and multilineage differentiation of human dental pulp cells (DPCs) remains unknown.* Methods*. To elucidate the functional role XPC played in pluripotency and multilineage differentiation of DPCs, expressions of XPC in DPCs with long-term culture were examined by real-time PCR and western blot. DPCs were transfected with lentiviral-mediated human XPC gene; then transfection rate was investigated by real-time PCR and western blot. Cell cycle, apoptosis, proliferation, senescence, multilineage differentiation, and expression of Oct-4/Sox2/c-Myc in transfected DPCs were examined.* Results*. XPC, Oct-4, Sox2, and c-Myc were downregulated at P7 compared with P3 in DPCs with long-term culture. XPC genes were upregulated in DPCs at P2 after transfection and maintained high expression level at P3 and P7. Cell proliferation, PI value, and telomerase activity were enhanced, whereas apoptosis was suppressed in transfected DPCs. Oct-4/Sox2/c-Myc were significantly upregulated, and multilineage differentiation in DPCs with XPC overexpression was enhanced after transfection.* Conclusions*. XPC plays an essential role in the modulation of pluripotency and multilineage differentiation of DPCs through regulation of Oct-4/Sox2/c-Myc.

## 1. Introduction

Dental pulp cells (DPCs), characterized by self-renewal, multilineage differentiation capability, and easy accessibility, are regarded as potential cell source for dental engineering [1]. Despite potential application in dental tissue regeneration, DPCs have been used for various degenerative diseases such as Alzheimer's disease, myocardial infarction, diabetes mellitus, bone defects, and spinal cord injuries [2]. However, it has been demonstrated that the progenitor/stem cell population was limited in DPCs, and primary cells with long-term* in vitro* culture were known to undergo replicative senescence, which limited pluripotency and restricted the potential application of DPCs in dental bioengineering [3]. Therefore, an investigation of the molecular mechanism to elevate the pluripotency of DPCs and optimize the cell characteristics would be of critical significance.

Previous studies revealed that overexpression of Oct-4, Sox2, Klf4, and c-Myc (OSKM) could induce mouse fibroblasts into induced pluripotent stem cells (iPSCs) with similar gene expression and developmental potential of mouse embryonic stem cells (mESCs) [4]. The endogenous expression level of Oct-4, Sox2, and c-Myc has been confirmed to be correlated with reprogramming and the pluripotency [4]. Oct-4 and Sox2, locating at the central of the transcriptional network, work cooperatively in maintaining pluripotency and inducing the reprogramming [5]. Recent study reported that a multisubunit stem cell coactivator complex (SCC) is required to activate the transcription of Oct-4/Sox2 and downstream target genes in ESCs. SCC is composed of the nucleotide excision repair (NER) proteins including xeroderma pigmentosum group C (XPC), RAD23B, and CETN2 [6]. XPC, as one component of the NER, serves as a transcriptional coactivator of DNA repair through modifying chromatin. XPC functions as critical factor modulating pluripotency, reprogramming, and genome integrity in mESCs through interacting with Oct-4 [7]. Lacking of XPC or RAD23B caused significant decrease of reprogramming to iPSCs, implying that the expression level of XPC may be related to the establishment efficiency of iPSCs [6].

In our previous study, we demonstrated that Oct-4, Sox2, and c-Myc were downregulated in DPCs with long-term* in vitro* culture, indicating that Oct-4 was closely related to the pluripotency of DPCs [8]. Considering that XPC interacts directly with Oct-4 and functions as critical factor modulating pluripotency, we established the XPC overexpression DPCs model by lentivirus transfection and extended our study to investigate the modulatory role of XPC played in cell proliferation and apoptosis and cell cycle and senescence and expression of pluripotency markers (Oct-4, Sox2, and c-Myc) and multilineage differentiation capability of DPCs during long-term* in vitro* culture. The data generated from this study will shed light on elevating the pluripotency of DPCs and their application in dental engineering.

## 2. Materials and Methods

### 2.1. Isolation and Expansion of Human DPCs

Normal human premolars and impact third molars were extracted from healthy young adults (12–28 years) undergoing orthodontic treatment at the Department of Oral and Maxillofacial Surgery, the Affiliated Stomatology Hospital of Sun Yat-sen University; informed consent was obtained from each patient. The protocol was approved by the University Ethic Committee. DPCs were obtained from human dental pulp tissues by explant culture as previously described [3]. DPCs were cultured in Dulbecco's modified Eagle medium with low glucose (DMEM-LG, Invitrogen, VIC, Australia) supplemented with 10% fetal bovine serum (FBS, HyClone, UT, USA), 10 U/mL penicillin G, and 10 mg/mL streptomycin (Invitrogen, VIC, Australia). DPCs were incubated at 37°C in 5% CO_2_. The medium was changed every 3 days.

### 2.2. Quantitative Real-Time Reverse Transcription-Polymerase Chain Reaction for the Expression of XPC, Oct-4, Sox2, and c-Myc in DPCs

Expression of XPC in DPCs from P1 to P7 was examined by real-time RT-PCR as previously described [3], while only the representative results of P3 and P7 were presented in Results. Total RNA of DPCs was isolated using Trizol reagent (Invitrogen, NY, USA) following the manufacturer's protocol. The concentration and quality of RNA samples were measured with spectrophotometers and gel electrophoresis. The first-strand cDNA was synthesized from 1 mg of total RNA using SuperScript III (Invitrogen, NY, USA) in a total volume of 20 mL. About 2.5 mL of the reaction mixture was incubated with SYBR Green I Master Mix (Applied Biosystems, NY, USA) in a total volume of 25 mL. The conditions for polymerase chain reaction (PCR) were as follows: 95°C for 10 min for activation, followed by 40 cycles of denaturation at 95°C for 15 s each, and, finally, primer extension at 60°C for 1 min.* XPC*,* Oct-4, Sox2, c-Myc,* and* 18s* mRNA were quantified using ABI Prism 7000 sequence detection system (Applied Biosystems, NY, USA). Each plate contained* 18s* as the housekeeping gene to normalize the PCR data. Primers used for detection were listed in Table 1. All experiments were repeated triplicate. Raw data were acquired to calculate the threshold cycle (Ct) value and relative gene expression values. The delta-delta Ct method was performed to analyze the results.

### 2.3. Western Blot for the Expression of XPC, Oct-4, Sox2, and c-Myc in DPCs

Western blot was performed as described previously [9]. Briefly, total protein of DPCs at P1 to P7 was measured by Bio-Rad Coomassie Blue protein assay (Bio-Rad Laboratories, Richmond, CA, USA), whereas only the representative results of P3 and P7 were presented in Results. Twenty micrograms of protein was diluted by 10% bromophenol blue and boiled before being separated by sodium dodecyl sulfate-polyacrylamide gel electrophoresis (SDS-PAGE) and transferred to a nitrocellulose membrane. The membranes were blocked in 5% low-fat milk at room temperature for 1 h, rinsed, and incubated with monoclonal antibodies mouse against human XPC (1 : 500 dilution, Abcam, MA, USA), Oct-4 (1 : 100, Chemicon, MA, USA), Sox2 (1 : 50, R&D System, MN, USA), c-Myc (1 : 50, Santa Cruz, CA, USA), or human GAPDH (1 : 1000 dilution, Santa Cruz, CA, USA) overnight at 4°C. After washing, the membrane was incubated with the HRP-conjugated secondary antibody (1 : 5000 dilution, Jackson, USA) at room temperature for 1 h. Immunoreactive proteins were then visualized by incubating membranes with electrogenerated chemiluminescence plus detection agents (GE Healthcare, USA).

### 2.4. Lentivirus Transfection of XPC in DPCs

The CDS region of XPC gene was amplified from a plasmid containing the full-length XPC sequence and cloned into Lentivector. After transformation into competent* E. coli*, the candidate clones were identified by PCR and sequencing. The constructed recombinant shuttle plasmid and lentivirus helper plasmid were cotransfected into HEK293T cells with the mediation of Lipofectamine 2000 and then propagated. A replication-deficient Lentivector, carrying the human XPC gene and the reporter gene, EGFP gene, was used in the current study. Forty-eight hours after transfection, EGFP expression in DPCs at passage 2 was observed by a fluorescence microscope (Axiovert, Zeiss, Germany).

### 2.5. Quantitative Real-Time PCR and Western Blot for Evaluation of XPC Expression in DPCs after Transfection

The expression of XPC in DPCs with XPC overexpression at P2, P3, and P7 was examined using real-time PCR and western blot 48 h after transfection as mentioned above. The nontransfected cells and cells transfected with vector served as control groups. Briefly, quantification of* XPC* and* 18s* mRNA was performed using ABI Prism 7000 sequence detection system (Applied Biosystems, NY, US). Primers used for detection were listed in Table 1. Delta-delta Ct method was performed to analyze the result. The total protein was measured by a Bio-Rad Coomassie Blue protein assay (Bio-Rad Laboratories, Richmond, CA, US). Proteins were detected with mouse monoclonal antibodies against human XPC (1 : 500, Abcam, MA, US) and GAPDH (1 : 1000 dilution, Santa Cruz Biotechnology, CA, US).

### 2.6. Cell Counting Kit 8 (CCK8) and Telomerase Activity Assay for Cell Proliferation and Senescence of DPCs with XPC Overexpression

Cell proliferation was measured by Cell Counting Kit 8 (CCK8) assay. DPCs of XPC overexpression group, vector group, and nontransfected group were grown in 96-well plates. The cells were cultured in DMEM supplemented with 10% FCS and 50 mg/mL gentamycin. Cell proliferation of DPCs at passages 3 and 7 at different time points (0, 3 d, 5 d, 7 d) was evaluated using the CCK8 (Dojindo, Tokyo, Japan), while only the representative results of 5 d were presented. 10 *μ*L of CCK8 solution was added to the culture medium and incubated for additional 3 h. The absorbance was determined at 450 nm wave length. Telomerase activity in DPCs from passages DPCs from passages 3 and 7 with/without XPC overexpression was detected using a quantitative telomerase detection kit (Allied Biotech, MD, US).

### 2.7. Flow Cytometry for Cell Cycle and Apoptosis

The cultures of DPCs were serum deprived for 24 h. 1 × 10^5^ DPCs of XPC overexpression group, vector group, and nontransfected group were harvested by trypsinization, washed twice in cold PBS, and fixed in 70% alcohol for 30 min on ice. After washing in cold PBS 3 times, cells were incubated with 0.5% propidium iodinate (PI) for 30 minutes at 4°C. Cells were analyzed using FACSCalibur flow cytometer (BD Biosciences, San Jose, CA, US). Data was analyzed using FCSExpress software.

### 2.8. Effect of XPC Overexpression on the Expression of Pluripotent Markers in DPCs

The expression of Oct-4, Sox2, and c-Myc on DPCs with XPC overexpression was investigated by immunofluorescent staining and real-time PCR. DPCs at P3 and P7 were cultured in chamber slides (Nunc, NY, USA) until 80% confluence and fixed with 3% paraformaldehyde for 15 min. The slides were washed in PBS 3 times, permeabilized with 0.1% Triton for 20 min, and incubated with 10% swine serum for 1 h. Slides were transferred to a humidified chamber and stained with XPC (1 : 400 dilution, Abcam, MA, USA), Oct-4 (1 : 400 dilution, Chemicon, MA, USA), Sox2 (1 : 400 dilution, R&D System, MN, USA), and c-Myc (1 : 400 dilution, Santa Cruz, CA, USA) antibodies overnight at 4°C. PBS was used as control. Samples were washed 3 times in PBS and incubated with a fluorochrome-labeled secondary antibody (1 : 100 dilution, Invitrogen, NY, USA) for 3 h. The sections were thoroughly washed in PBS and mounted for fluorescent microscopic analysis (Axiovert, Zeiss, Germany). Real-time PCR was performed as mentioned above. Total RNA of DPCs at P3 and P7 was isolated by Trizol reagent. Primers used for detection were listed in Table 1. Each plate contained* 18s* as the housekeeping gene to normalize the PCR data. Delta-delta Ct method was performed to analyze the result.

### 2.9. Effect of XPC Overexpression on Multilineage Differentiation of DPCs

For odontogenic differentiation, DPCs of XPC overexpression group, vector group, and nontransfected group were odontogenically induced in growth medium containing 10 mM *β*-glycerophosphate (Sigma-Aldrich, Australia), 50 *μ*M ascorbic acid (Sigma-Aldrich, Australia), and 100 nM dexamethasone (Sigma-Aldrich, Australia) for 21 d. The expression of DSPP was explored by immunofluorescent staining as mentioned above. DPCs were stained with DSPP antibody (1 : 400 dilution, Chemicon, MA, USA). The mRNA expression of* DMP1* and* DSPP* was evaluated by real-time PCR. Primers used for detection are listed in Table 2.

For adipogenic differentiation, DPCs were incubated in the adipogenic induction medium consisting of 0.5 mM 3-isobutyl-1-methylxanthine (IBMX, Sigma, USA), 10 *μ*g/mL insulin (GIBCO, USA), 1 mM dexamethasone, 100 *μ*M indomethacin (Sigma, USA), and 15% FBS in *α*-MEM for 3 d, followed by the adipogenic maintenance medium consisting solely of 10 *μ*g/mL insulin and 15% FBS for 1 d. After completing the three cycles of induction and maintenance, the induced cells were incubated for another 14 d in adipogenic maintenance medium. Adipogenic differentiation was confirmed by immunofluorescent staining and the mRNA expression of* LPL* and* PPARγ2* was confirmed by real-time PCR. DPCs were stained with LPL antibody (1 : 400 dilution, Chemicon, MA, USA).

For chondrogenic differentiation, DPCs were chondrogenically inducted by culturing in high cell density through pelletation (2 × 10^5^ cells per pellet) in 500 *μ*L chondrogenic differentiation medium. Serum-free chondrogenic differentiation medium consisted of high glucose DMEM supplemented with 10 ng/mL of transforming growth factor-*β*3 (TGF-*β*3, R&D Systems, USA), 10 nM dexamethasone, 50 mg/mL of ascorbic acid, 10 mg/mL of sodium pyruvate (Sigma, USA), 10 mg/mL of proline (Sigma, USA), and an insulin-transferrin-selenium supplement. Pellets were allowed to differentiate under 3-dimensional conditions in 15 mL centrifuge tubes at 2% or 20% O_2_ tension. After 21 d of chondrogenic differentiation, the pellets were fixed with 4% PFA and embedded in paraffin. Expression of collagen type II was examined by immunofluorescent staining and real-time PCR. DPCs were stained with collagen type II antibody (1 : 400 dilution, Chemicon, MA, USA).

### 2.10. Statistical Analysis

All experiments were repeated at least three times. The SPSS19.0 software package (SPSS Inc., Chicago, IL) was used for the statistical tests. All the data were analyzed using one-way ANOVA analysis and Student's *t*-test. The difference was considered as being of statistical significance at *p* < 0.05.

## 3. Results

### 3.1. Expression of XPC, Oct-4, Sox2, and c-Myc in DPCs at Various Passages

**Figure 1:**
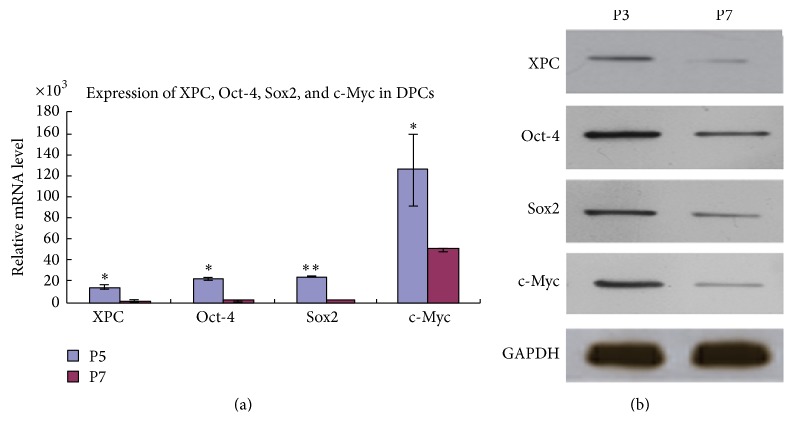
Expression of* XPC, Oct-4*,* Sox2,* and* c-Myc* in DPCs at various passages. Real-time PCR showed that mRNA expression of* XPC, Oct-4*,* Sox2,* and* c-Myc* was significantly higher in DPCs at passage 3 compared with passage 7 (a) (^*∗*^ *p* < 0.05, ^*∗∗*^ *p* < 0.001). Western blot showed that the protein expression of XPC, Oct-4, Sox2, and c-Myc revealed similar expression pattern with real-time PCR, which was stronger in DPCs at passage 3 compared with passage 7 (b).

Expression of XPC in DPCs from passages 1 to 7 were investigated by real-time PCR and western blot, while only the results of representative passages (P3 and P7) were presented. Real-time PCR (Figure 1(a)) and western blot (Figure 1(b)) indicated that the mRNA and protein expression of XPC, Oct-4, Sox2, and c-Myc showed similar expression pattern, which was significantly downregulated at P7 compared with P3 (^*∗*^ *p* < 0.05, ^*∗∗*^ *p* < 0.001).

### 3.2. Gene Transfection and Expression of XPC in DPCs

**Figure 2:**
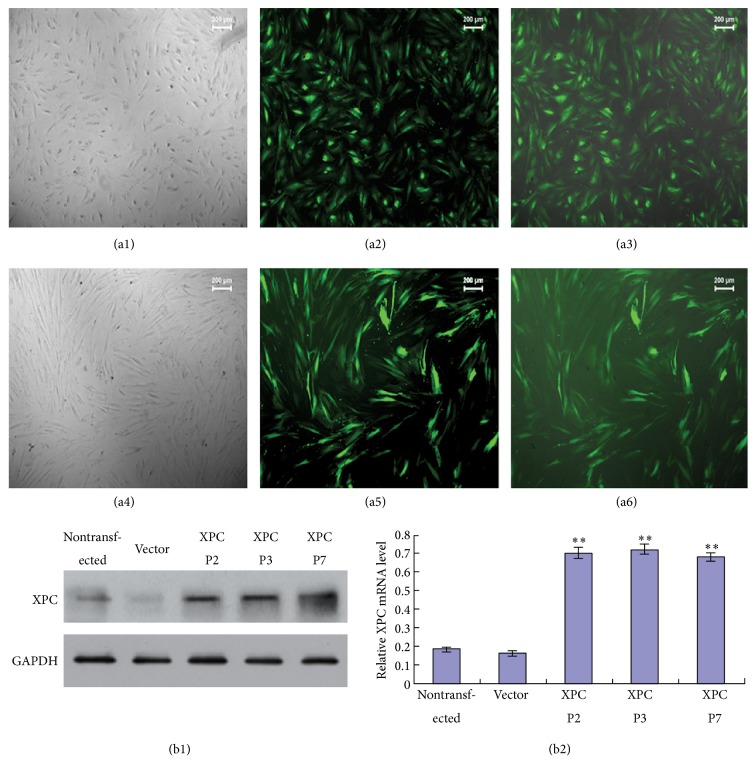
Establishment of XPC overexpression DPCs model. Expression of pCDH-CMV-XPC-EF1-copGFP plasmid in HEK293T cells and DPCs at P2 (a1–a6). Strong green fluorescence was detected in HEK293T cells (a2, ×50) and DPCs (a5, ×100) after transfection. Combined figures of the bright field and fluorescence figure (a3, a6). Western blot and real-time PCR showed that the expression of XPC was enhanced in XPC+/DPCs at P2 at both protein and mRNA level compared with vector group, and XPC maintained high expression level in DPCs at P3 and P7 (^*∗∗*^ *p* < 0.01) (b1, b2).

Expression of pCDH-CMV-XPC-EF1-copGFP plasmid in HEK293T cells and DPCs was shown in Figure 2. Strong green fluorescence could be viewed in HEK293T cells after transfection (Figure 2(a2)). All DPCs with XPC overexpression showed green fluorescent staining (Figure 2(a5)). The result of real-time PCR revealed that XPC mRNA was significantly higher in XPC transfected DPCs at P2, P3, and P7 compared with the vector group (Figure 2(b2), ^*∗∗*^ *p* < 0.001), whereas there is no significant difference between nontransfected and vector groups (*p* > 0.05). Western blot (Figure 2(b1)) demonstrated that the protein expression of XPC was strongly upregulated in DPCs at P2, P3, and P7 after transfection, which agreed with the result of real-time PCR (Figure 2(b2)). These results indicated that the XPC overexpression DPCs model was successfully established, which could be passaged and XPC maintained consistently high expression level in transfected DPCs even after long-term* in vitro* culture.

### 3.3. Cell Proliferation, Cycle, and Apoptosis of DPCs with XPC Overexpression

**Figure 3:**
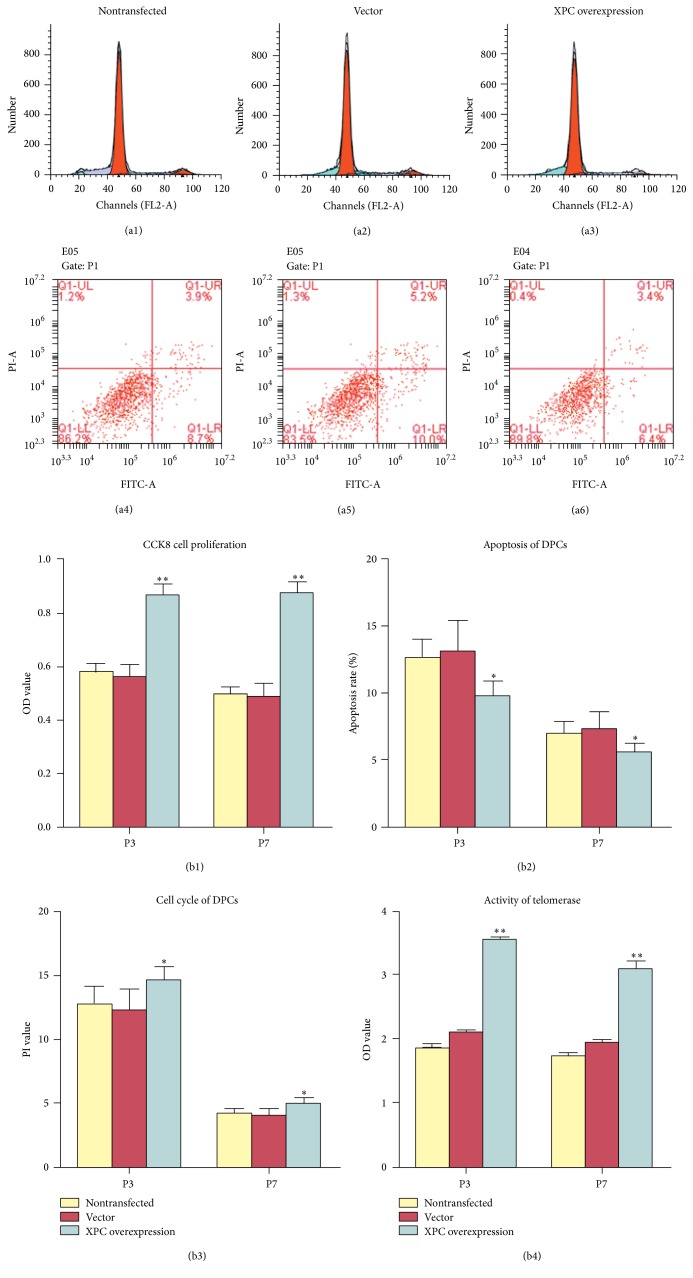
Effect of XPC on cell proliferation and apoptosis and cell cycle and senescence of DPCs with* in vitro* culture. CCK8 revealed that cell proliferation of transfected DPCs at both P3 and P7 was significantly enhanced (b1, ^*∗∗*^ *p* < 0.001). The result of FACS (a1–a6) demonstrated that the cell number of transfected DPCs at P3 and P7 in G0/G1 was significantly downregulated compared with the vector groups; DPCs were arrested in G2/M and S phase. The percentage of PI = (S + G2/M) value (b3, ^*∗*^ *p* < 0.05) and telomerase activity (b4, ^*∗∗*^ *p* < 0.001) of transfected DPCs at both P3 and P7 was significantly upregulated, albeit the apoptosis rate was significantly downregulated (b2, ^*∗*^ *p* < 0.05).

To investigate the biological function of XPC in DPCs, CCK8 and FACS were applied to DPCs with XPC overexpression. CCK8 revealed that cell proliferation of transfected DPCs at both P3 and P7 was significantly enhanced (Figure 3(b1), ^*∗∗*^ *p* < 0.001), whereas there is no significant difference between control group and vector group (*p* > 0.05). The result of FACS demonstrated that the cell number of transfected DPCs at P3 and P7 in G0/G1 was significantly downregulated compared with the vector groups; DPCs were arrested in G2/M and S phase. The percentage of PI = (S + G2/M) value and telomerase activity of transfected DPCs at both P3 and P7 was significantly upregulated, albeit the apoptosis rate was significantly downregulated (Figures 3(b2)–3(b4), Table 3, *p* < 0.05). Therefore, XPC could enhance cell proliferation and telomerase activity of DPCs, arrested cells in G2/M and S phase, and upregulated PI value, albeit suppressed apoptosis, suggesting the role XPC played in the maintenance of pluripotency in DPCs.

### 3.4. Expression of Oct-4, Sox2, and c-Myc in DPCs with XPC Overexpression

**Figure 4:**
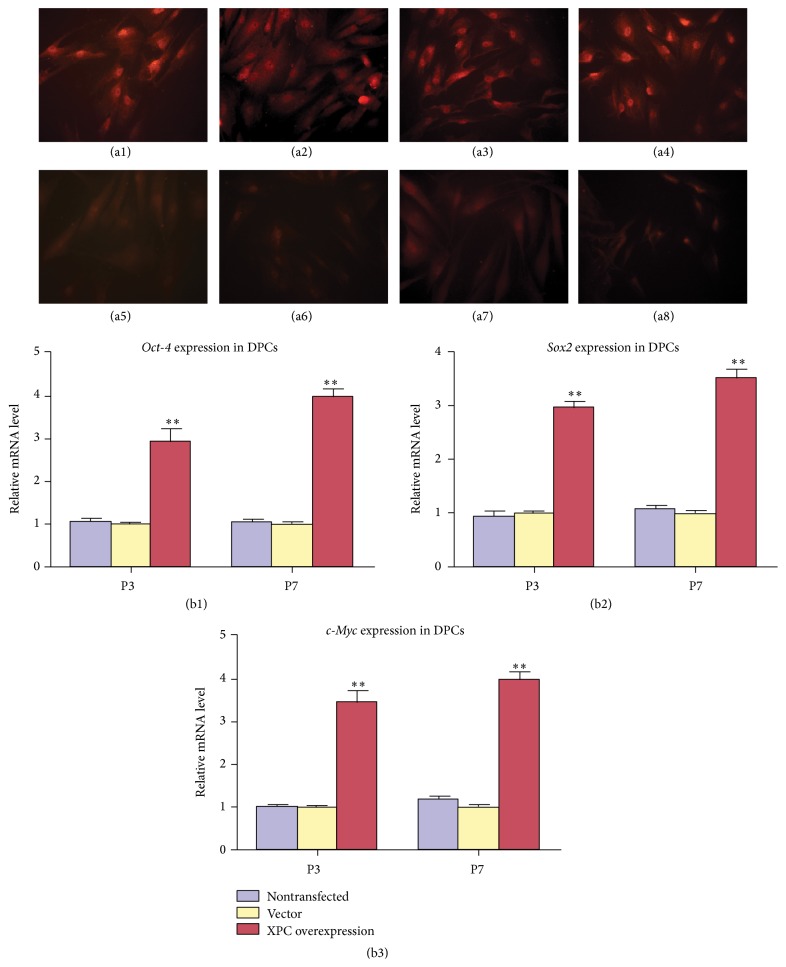
Effect of XPC on the expression of Oct-4, Sox2, and c-Myc in DPCs at various passages. Immunofluorescent staining revealed that XPC (a1), Oct-4 (a2), Sox2 (a3), and c-Myc (a4) were strongly expressed in DPCs with XPC overexpression at P7, mainly located in nucleus and moderately expressed in cytoplasm, whereas XPC (a5), Oct-4 (a6), Sox2 (a7), and c-Myc (a8) were barely detected in DPCs at P7 without transfection. Real-time PCR indicated that the mRNA expression of* XPC, Oct-4, Sox2*, and* c-Myc* was upregulated significantly in DPCs with XPC overexpression at both P3 and P7 compared with vector groups (^*∗∗*^ *p* < 0.001). There is no significant difference between nontransfected group and vector group (*p* > 0.05) (b1–b3).

Real-time PCR indicated that the mRNA level of* Oct-4, Sox2,* and* c-Myc* was significantly upregulated in DPCs with XPC transfection at both P3 and P7 (Figures 4(b1)–4(b3), ^*∗∗*^ *p* < 0.001). Similarly, immunofluorescent staining revealed that XPC (Figure 4(a1)), Oct-4 (Figure 4(a2)), Sox2 (Figure 4(a3)), and c-Myc (Figure 4(a4)) were strongly expressed in DPCs with XPC overexpression at P7, mainly located in nucleus and moderately expressed in cytoplasm. Whereas XPC (Figure 4(a5)), Oct-4 (Figure 4(a6)), Sox2 (Figure 4(a7)), and c-Myc (Figure 4(a8)) were barely detected in DPCs at P7 without transfection. Oct-4, Sox2, and c-Myc showed extensive expression in DPCs at P3 with/without XPC expression (data not shown).

### 3.5. The Multilineage Differentiation of DPCs with XPC Overexpression

**Figure 5:**
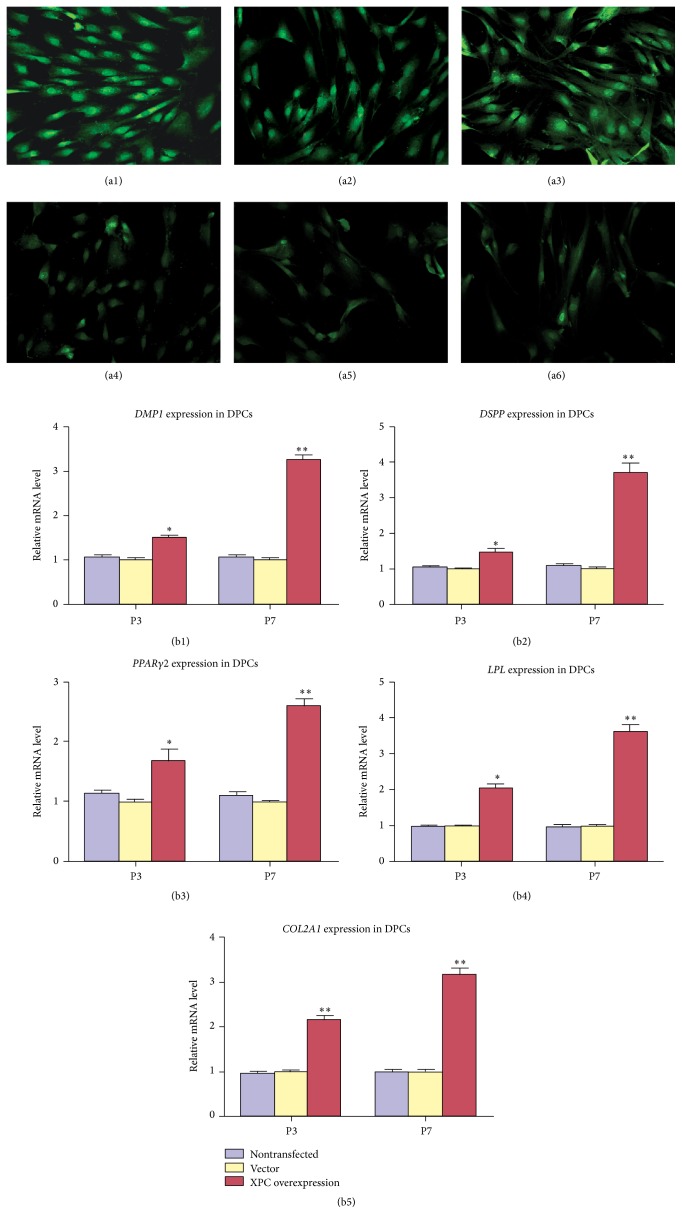
Effect of XPC on the multilineage differentiation capability of DPCs after transfection. After 21 d of multilineage differentiation induction, immunofluorescent staining indicated that DSPP (a1), LPL (a2), and collagen type II (a3) were extensively expressed in DPCs at P7 with XPC overexpression, strongly expressed in the nucleus and moderately expressed in the cytoplasm of DPCs. However, DSPP (a4), LPL (a5), and collagen type II (a6) revealed weak expression in the nucleus and cytoplasm of DPCs at P7 without transfection. Real-time PCR indicated that mRNA expression level of odontogenic markers (*DMP1, DSPP*), adipogenic markers (*PPARγ2, LPL*), and chondrogenic markers (*collagen type II*) increased significantly in DPCs at P3 and P7 with XPC overexpression compared with vector groups (b1–b5).

The odontogenic/adipogenic/chondrogenic differentiation capability of DPCs with XPC overexpression at P7 was investigated by immunofluorescent staining (Figures 5(a1)–5(a6)), and the mRNA expression of lineage related genes in DPCs at P3 and P7 was further confirmed by real-time PCR. After odontogenic/adipogenic/chondrogenic induction, immunofluorescent staining indicated that DSPP (Figure 5(a1)), LPL (Figure 5(a2)), and Collagen type II (Figure 5(a3)) were extensively expressed in DPCs at P7 with XPC overexpression, strongly expressed in the nucleus and moderately expressed in cytoplasm. However, the expression of DSPP (Figure 5(a4)), LPL (Figure 5(a5)), and Collagen type II (Figure 5(a6)) was barely detected in the DPCs at P7 without XPC transfection. Real-time PCR revealed that odontogenic markers (*DMP1, DSPP*), adipogenic markers (*LPL*,* PPARγ2*), and chondrogenic marker (*Collagen* type II) showed similar expression pattern, upregulated in DPCs with XPC overexpression at both P3 and P7 compared with the vector groups (Figures 5(b1)–5(b5), ^*∗*^ *p* < 0.05, ^*∗∗*^ *p* < 0.01). Therefore, XPC could effectively enhance the multilineage differentiation capability of DPCs with long-term* in vitro* culture.

## 4. Discussion

Somatic cells could be reprogrammed to iPSCs with ESCs-like characteristics through introduction of Oct-4, Sox2, c-Myc, Klf4, and Nanog [4]. Oct-4 is one of the key factors regulating pluripotency and reprogramming. Oct-4 expression at the optimized level is necessary for self-renewal of mESCs [10]. The identification of Oct-Sox2 composite consensus sequence confirmed that Oct-4 activates transcription of the pluripotency-associated transcription factors (TFs) in the heterodimer through direct cooperation with Sox2. Oct-4/Sox2 complexes are key transcriptional activators to recognize the specific DNA cis-acting elements in reprogramming markers [5]. The Oct-Sox2* cis*-control element is a common configuration in the promoters of Oct-4/Sox2-activated genes, through which Oct-4 and Sox2 cooperate with various cofactors to activate the pluripotency network and initiate the reprogramming process [11, 12]. Although Oct-4-Sox2 complexes are at the top of the regulatory hierarchy, some studies indicated that even the coexpression of Oct-4 and Sox2 was not sufficient to activate the Nanog promoter reporter in 293 or NIH3T3 cells [12]. Recent study reported that SCC is required to activate the transcription of Oct-4/Sox2 and downstream target genes in ESCs. SCC could maintain and reestablish stem cell pluripotency through functioning as a transcriptional coactivator of Oct-4/Sox2. SCC interacts directly with Oct-4/Sox2 and is recruited to the Oct-4 promoter AID genomic regions occupied by Oct-4/Sox2 [6]. Depletion of SCC/XPC resulted in reduction of pluripotency in ESCs and reprogramming into iPSCs, implying that loss of the SCC/XPC complex decreased transcriptional integrity and the ability of pluripotency reestablishment. Thus SCC/XPC is required for the modulation of self-renewal and reprogramming efficiency [6, 12]. SCC/XPC is also involved in the process of chromatin reorganization and alternation of the epigenetic landscape for iPSCs conversion [13].

XPC, as one component of the NER, functions as a transcriptional coactivator independently of DNA repair through modifying chromatin. XPC is the essential element to recognize DNA distortions and recruit the basal transcription factors, like TFIIH [14]. XPC trimeric complex is required to activate the Oct-4/Sox2 mediated transcription for the maintenance and reestablishment of ESCs specific gene expression [6, 14]. Removal of the C-terminal region of XPC, including the interaction sites with Rad23 and Cetn2, resulted in alternations of the gene expression profile and pluripotency of ESCs [7]. Downregulation of XPC and RAD23B could significantly decrease Oct-4/Sox2 dependent transcription of Nanog, indicating that SCC/XPC is closely associated with pluripotency [12]. XPC mainly binds to RAD23B, which stabilizes XPC and effectively binds XPC to DNA lesions. Simultaneous knock-down of XPC, RAD23B, and CETN2 resulted in decrease in expression of Oct-4 and self-renewal of ESCs [12]. XPC silencing resulted in decreased amount of CENTRIN2 expression, which affected both its centrosomal and nuclear localization. Thus XPC deficiency indirectly slows down cell division and lower DNA repair capacity, confirming the essential role XPC played in CENTRIN2's internalization into the nucleus of human cells, DNA repair, and cell survival [15].

In our previous study, we revealed that the mRNA and protein expression of Oct-4, Sox2, and c-Myc in DPCs were firstly upregulated and peaked at passage 2 and then they were subsequently downregulated afterwards [8]. In the present study, real-time PCR and western blot indicated that XPC showed a similar expression pattern with Oct-4, Sox2, and c-Myc, suggesting that XPC might work cooperatively with Oct-4, Sox2, and c-Myc in the maintenance and reestablishment of pluripotency in DPCs with* in vitro* culture. Moreover, Oct-4, Sox2, and c-Myc significantly increased in DPCs with XPC overexpression. These results agreed with previous reports that XPC is required to activate the Oct-4/Sox2 mediated maintenance and reestablishment of pluripotency through interacting with downstream transcription factors [6, 12].

Prolonged* in vitro* culture has been reported to cause morphologic alternations and decrease expression of pluripotent markers and cell senescence [16, 17]. Oncogenic stress, DNA damage and stress stimulation, and so forth are contributory factors to cell senescence. Accumulation of DNA deletions or mutations can result in aging and senescence [16]. DNA damage with genomic defect or mutation could stimulate oncogenes and inactivate tumor suppressor genes, causing accelerate aging, loss of homeostasis, and tumorigenesis [17]. DNA repair is essential to the homeostasis and genomic stability of all organisms [18]. Cell cycle arrest could prevent mutagenesis and DNA damage [19]. NER factors are related to cell cycle checkpoints, which control apoptosis, cell cycle arrest, and DNA repair and enhance genomic stability and cell survival [20]. It has been shown that oxidative DNA damage accumulation increased without presence of XPC. XPC is involved in the* in vivo* and* in vitro* initiation of DNA damage-induced biological process, including removal of oxidative DNA damage, redox homeostasis, and cell cycle regulation [14, 21]. Persistent DNA damage without XPC resulted in sustaining telomere attrition; thus XPC is required to maintain telomere length, integrity, and stability. Failure of DNA repair and telomere shortening result in replicative senescence or cell death [22].

In the present study, significant upregulation of cell proliferation, telomerase activity, and multilineage differentiation was observed in DPCs with XPC overexpression, while apoptosis was restrained in DPCs with XPC overexpression. Moreover, we observed an increase in the percentage of G2/M and S phase cells in DPCs after XPC transfection. Considering that XPC plays dual roles in transcriptional coactivation and DNA repair to maintain pluripotency in ESCs [6, 12, 15], the result from present study implied that XPC might modulate cell senescence and pluripotency through cell cycle regulation. It might be possible that XPC increases pluripotency of DPCs through elimination of damaged cells, subsequently reducing the risk of cell cycle reentry and acquisition of mutations. These results agreed with the previous reports and expanded our data regarding the effect of XPC on cell senescence and pluripotency in dental derived cells [6, 12, 15, 22].

## 5. Conclusions

Taken together, the present study demonstrated that pluripotency could be reachieved in DPCs at late passages with XPC overexpression through regulation of Oct-4/Sox2/c-Myc network; therefore XPC may play an essential role in the modulation of pluripotency and multilineage differentiation of DPCs via regulation of Oct-4/Sox2/c-Myc. The knowledge generated from this study will offer better understanding on the potential application of DPCs in dental tissue engineering and improving the method to induce pluripotent cells.

## Figures and Tables

**Table 1:** Primer sequences used in quantitative real-time polymerase chain reaction.

Gene	Primers
XPC	Forward: 5′-TGAGACCATACCAGAGCCCA-3′
	Reverse: 5′-GGGCATACAGAGGGTGGTTC-3′

Oct-4	Forward: 5′-GCT CGA GAA GGA TGT GGT C-3′
	Reverse: 5′-ATC CTC TCG TTG TGC ATA GTC G-3′

Sox2	Forward: 5′-GAGAACCCCAAGATGCACAAC-3′
	Reverse: 5′-CGCTTAGCCTCGTCGATGA-3′

c-Myc	Forward: 5′-GGCTCCTGGCAAAAGGTCA-3′
	Reverse: 5′-AGTTGTGCTGATGTGTGGAGA-3′

18s	Forward: 5′-CCTGGATACCGCAGCTAGGA-3′
	Reverse: 5′-GCGGCGCAATACGAATGCCCC-3′

**Table 2:** Primer sequences used in quantitative real-time polymerase chain reaction.

Gene	Primers
DMP1	Forward: 5′-CTGCAACACAGG GAAATGGA-3′
	Reverse: 5′-ACGGACACTGCTC CATCCTT-3′

DSPP	Forward: 5′-ATT CCG GTT CCC CAG TTA GTA-3′
	Reverse: 5′-CTG TTG CTA GTG GTG CTG TT-3′

PPAR*γ*2	Forward: 5′-CTT CGG AAT CAG CTC TGT GGA C-3′
	Reverse: 5′-GCA TCC TTC ACA AGC ATG GAC T-3′

LPL	Forward: 5′-GGG AGT TTG GCT CCA GAG TTT-3′
	Reverse: 5′-TGT GTC TTC AGG GGT CCT TAG-3′

Col2a1	Forward: 5′-TCC CAG AAC ATC ACC TAC CAC T-3′
	Reverse: 5′-GGT CTT CTG TGA TCG GTA CTC G-3′

18s	Forward: 5′-CCTGGATACCGCAGCTAGGA-3′
	Reverse: 5′-GCGGCGCAATACGAATGCCCC-3′

**Table 3:** Cell cycle of DPCs with long-term *in vitro* culture after XPC transfection.

Groups	G0/G1	G2/M	S	PI = (G2/M + S)%
Nontransfected P3	87.1 ± 1.5	8.04 ± 1.4	4.76 ± 1.5	12.8 ± 1.5
Vector P3	87.6 ± 1.6	7.92 ± 1.5	4.28 ± 1.5	12.3 ± 1.6
XPC P3	85.2 ± 1.1^*∗*^	8.97 ± 1.5^*∗*^	5.73 ± 1.5^*∗*^	14.7 ± 1.1^*∗*^
Nontransfected P7	95.8 ± 0.4	2.04 ± 0.4	2.05 ± 0.4	4.09 ± 0.4
Vector P7	95.9 ± 0.4	2.01 ± 0.4	2.03 ± 0.4	4.04 ± 0.4
XPC P7	94.9 ± 0.4^*∗*^	3.12 ± 0.4^*∗*^	1.90 ± 0.4^*∗*^	5.02 ± 0.4^*∗*^

(Mean ± SD%  *N* = 3).

^*∗*^ *P* < 0.05.
